# Phosphine Exposure Among Emergency Responders — Amarillo, Texas, January 2017

**DOI:** 10.15585/mmwr.mm6713a2

**Published:** 2018-04-06

**Authors:** Emily M. Hall, Ketki Patel, Kerton R. Victory, Geoffrey M. Calvert, Leticia M. Nogueira, Heidi K. Bojes

**Affiliations:** ^1^Texas Department of State Health Services; ^2^Emergency Preparedness and Response Office, National Institute for Occupational Safety and Health, CDC; ^3^World Trade Center Health Program, National Institute for Occupational Safety and Health, CDC.

Phosphine is a highly toxic gas that forms when aluminum phosphide, a restricted-use pesticide* typically used in agricultural settings, reacts with water. Acute exposure can lead to a wide range of respiratory, cardiovascular, and gastrointestinal symptoms, and can be fatal (*1*). On January 2, 2017, the Texas Department of State Health Services (DSHS) was notified by the Texas Panhandle Poison Center of an acute phosphine exposure incident in Amarillo, Texas. DSHS investigated potential occupational phosphine exposures among the 51 on-scene emergency responders; 40 (78.4%) did not use respiratory protection during response operations. Fifteen (37.5%) of these 40 responders received medical care for symptoms or as a precaution after the incident, and seven (17.5%) reported new or worsening symptoms consistent with phosphine exposure within 24 hours of the incident. Emergency response organizations should ensure that appropriate personal protective equipment (PPE) is used during all incidents when an unknown hazardous substance is suspected. Additional evaluation is needed to identify targeted interventions that increase emergency responder PPE use during this type of incident.

## Investigation and Response

At approximately 5:00 a.m. on January 2, 2017, emergency responders were dispatched to a single-family residence following a 9-1-1 call reporting shortness of breath, loss of consciousness, and other symptoms among occupants. These health effects were initially thought to be the result of carbon monoxide exposure; however, air monitoring detected no carbon monoxide. Emergency responders discovered that a restricted-use pesticide containing aluminum phosphide had been applied outside the residence several days before the 9-1-1 call. It was determined that phosphine had been released when the pesticide reacted with water, first from ambient humidity, and then when attempts were made to wash the pesticide away on January 1, 2017.

Because a hazardous substance was suspected, the City of Amarillo dispatched a hazardous materials (HAZMAT) team composed of fire department personnel and established a secure perimeter around the home. Persons found inside were assisted out of the residence, given emergency medical care, and transported to a nearby hospital. Domestic animals found on-scene were decontaminated by dry brushing and taken to a local animal welfare facility. The local health authority issued a health alert to inform medical care providers.

Later on January 2, the City of Amarillo requested a toxicologic consultation from DSHS related to the incident. Based on incident response activities described during the consultation, it was determined that emergency responders might have been exposed to phosphine at the scene. Therefore, DSHS investigated potential occupational phosphine exposures and associated health effects among all City of Amarillo personnel who participated in the emergency response.

DSHS reviewed Texas Poison Control Network call records related to the event, and then designed a standardized health questionnaire based on the Agency for Toxic Substances and Disease Registry’s (ATSDR’s) Assessment of Chemical Exposures toolkit to interview potentially exposed emergency responders (*2*). Data collected included demographics, work history, role in the response, PPE use, potential exposure to phosphine and related acute health effects, emergency response training, and medical care received. Local health department personnel administered the questionnaire for DSHS via in-person and telephone interviews from January 23 through February 3, 2017. Data were analyzed by DSHS; data that could potentially identify an individual were suppressed if counts were fewer than five.

Fifty-one emergency responders participated on-scene in the response. Air monitoring data were limited, so all were considered potentially exposed to phosphine and contacted for a follow-up interview. All 51 (100%) responders participated, including fire, police, animal welfare, and emergency medical services personnel. The median emergency responder age was 31 years (range = 20–54 years) and the median length of time in their current job was 5 years (range = 2 months–30 years).

Eleven responders (21.6%), including seven firefighters and HAZMAT team members, reported use of respiratory protection while on-scene; none of these persons reported symptoms within 24 hours or sought medical care following the incident (Table 1). Fifteen (37.5%) of the 40 emergency responders who did not use respiratory protection received medical care for symptoms or as a precaution after the incident. Seven (17.5%) of these 40 reported new or worsening symptoms within 24 hours of the response. Symptoms included irritability, ocular pain or burning, headache, nausea, drowsiness, dizziness, burning of nose or throat, abdominal cramps, diarrhea, generalized weakness, trembling legs or hands, and trouble walking.

**TABLE 1:** Characteristics of emergency responders potentially exposed during a phosphine release event (n = 51) — Amarillo, Texas, 2017

Characteristic	No.* (%)
**Role during response operations^†^**	
Provide medical care	15 (29.4)
Animal control	9 (17.6)
Rescue victims/First response	9 (17.6)
HAZMAT team	8 (15.7)
Security/Guard perimeter	5 (9.8)
Supervise	5 (9.8)
Operations and logistics	<5 (—)
Other	<5 (—)
Unknown	<5 (—)
**Initial information received before on-scene arrival^†^**	
Medical emergency	38 (74.5)
Possible carbon monoxide release	11 (21.6)
Unknown chemical hazard	10 (19.6)
HAZMAT	7 (13.7)
Phosphine release	<5 (—)
Other	<5 (—)
Unknown/Missing	<5 (—)
**Hours worked at incident site** ^§^	
<1	15 (30.0)
1–1.9	17 (34.0)
2–2.9	7 (14.0)
≥3	11 (22.0)
**Respiratory protection used**	
Yes	11 (21.6)
No	40 (78.4)
**Symptoms of illness within 24 hours of the incident^¶^**	
Yes	7 (13.7)
No or not sure	44 (86.3)
**Medical care sought**	
Yes	15 (29.4)
No	36 (70.6)

**Abbreviation:** HAZMAT = hazardous materials.

* Counts <5 suppressed to protect confidentiality.

^†^ Categories are not mutually exclusive.

^§^ n = 50.

^¶^ Fifteen (37.5%) of the 40 emergency responders who did not use respiratory protection received medical care for symptoms or as a precaution after the incident. Seven (17.5%) of these 40 reported new or worsening symptoms within 24 hours of the response. None of the 11 who used respiratory protection reported symptoms or having received medical care.

Among the 40 responders who did not use respiratory protection, 14 (35%) provided the following nonmutually exclusive reasons: did not know it was needed or were not told to use it (five); rescuing victims was more important (four); did not know the contaminant was present (four); was not required for the work performed (two); and did not have equipment (one).

Thirty-seven (72.5%) of the 51 responders stated that their agency had plans or standard operating procedures for responding to situations where hazardous materials are present. Forty (78.4%) reported receiving at least one emergency response training^†^ before the incident (Table 2), including 29 (72.5%) of the 40 responders who did not use respiratory protection.

**TABLE 2:** Emergency response trainings received by responders who were potentially exposed during a phosphine release event (n = 51) — Amarillo, Texas, 2017

Training	No.* (%)
Any emergency response training^†^	40 (78.4)
First responder awareness	27 (52.9)
Hazardous materials technicians, 24 hr.	26 (51.0)
First responder operations, 8 hr.	15 (29.4)
Other^§^	14 (27.5)
HAZWOPER, 24 hr.	5 (9.8)
HAZWOPER, 40 hr.	<5 (—)
No emergency response training^¶^	11 (21.6)

**Abbreviation:** HAZWOPER = hazardous waste operations and emergency response.

* Counts <5 suppressed to protect confidentiality.

^†^ Categories are not mutually exclusive.

^§^ Includes animal control, animal cruelty training (levels 1, 2, 3); National Incident Management Incident Command System 100, 200, 300, 400, 700 and 800; and police academy training.

^¶^ Responders might not have been required to take trainings listed as a condition of employment.

## Discussion

CDC and other agencies have developed protocols and tools to facilitate implementation of best practices for responding to incidents involving unknown chemical hazards, and their use has been recommended following similar incidents in the past (*3*,*4*). Federal regulations require the use of appropriate respiratory protection in emergency responses involving suspected hazardous substances.^§^ DSHS recommends implementation of these recommendations and has worked with the National Institute for Occupational Safety and Health Emergency Preparedness and Response Office to develop and disseminate educational materials targeted to emergency responders and emergency response organizations to highlight the importance of using appropriate respiratory protection.

The 51 emergency responders involved in this incident were faced with limited information about the hazards present, combined with the need to act quickly to rescue victims. Many did not use recommended respiratory protection. These issues exemplify challenges faced by emergency responders who often confront unknown hazards and, given the need to save lives or secure the scene, might feel they do not have time to identify, obtain, and don recommended PPE (*3*,*6*). They also might perceive that PPE would physically restrict their ability to perform required tasks (*6*).

Studies of other incidents involving the known or suspected release of hazardous substances have similarly found low prevalences of respiratory PPE use among emergency responders, especially police and emergency medical services. For example, one investigation found that among 92 emergency personnel who responded to an unintentional vinyl chloride release, only 20 (21.7%) reported using indicated respiratory protection during the response (*3*). Multiple studies have found that the prevalence of appropriate respiratory protection was low among emergency responders to the World Trade Center collapse (*7*). A recent analysis of ATSDR surveillance data found that, among 1,275 emergency personnel with known PPE status who were injured or became ill during acute hazardous substance release incident responses during 2002–2012, only 382 (30.0%) wore some type of respiratory protection (*8*). Respiratory protection prevalence was 45.8% among injured firefighters, compared with 1.4% among police and 2.3% among emergency medical services personnel. Firefighters’ injuries were more likely to involve trauma or burns than were those sustained by other types of responders. Because PPE use among emergency personnel who were not injured or ill was not collected, it was not possible to assess the effectiveness of PPE in preventing injuries and illness.

The findings in this report are subject to at least two limitations. First, information bias is possible because exposure and symptom status were identified by self-report. However, no data were available to estimate individual phosphine exposure. Personal air monitoring was not conducted, and air samples were not collected inside the residence before remediation. Second, not all symptomatic persons sought medical treatment, so medical records were insufficient to assess health outcomes. Therefore, self-report was the most comprehensive source of information on exposure and health outcomes.

This incident demonstrates that, although important, standard emergency responder trainings alone might not ensure correct PPE use during this type of incident response. Studies among health care, farm, construction, and manufacturing workers have found that individual behavioral interventions (e.g., training and education) alone do not significantly improve respiratory protection use (*9*). Some studies have found that interventions targeting social and organizational factors, such as safety climate, do positively impact PPE use (*6*). However, few studies of PPE-related behavioral interventions have been conducted among emergency responders, so methods for improving compliance with existing PPE guidance and regulations among responders are not well understood. Additional evaluation is needed to identify targeted individual and organizational interventions that effectively increase appropriate PPE use among emergency responders during incidents involving unknown hazards.

**Summary**

**What is already known on this topic?**

To prevent exposure to harmful chemical substances among emergency responders, use of respiratory and other personal protective equipment (PPE) is recommended during incident responses when release of an unknown hazardous substance is suspected. Past studies have found low prevalences of respiratory protection use during hazardous substance release incidents.

**What is added by this report?**

Forty (78.4%) of 51 emergency personnel responding to an acute phosphine exposure incident in Texas in January 2017 did not use respiratory protection, including 15 (37.5%) who received medical care after the incident and seven (17.5%) who reported new or worsening symptoms consistent with phosphine exposure within 24 hours of the incident. The majority had received standard emergency response training and knew of agency standard operating procedures for responding to incidents involving hazardous substances.

**What are the implications for public health practice?**

Although emergency responder risk of exposure during incidents involving unknown hazardous substances is well documented, methods for improving compliance with existing recommendations and regulations for respiratory protection use are not well understood. Additional evaluation is needed to identify targeted interventions that effectively increase appropriate PPE use among emergency responders during incidents involving such unknown hazards.

## Footnotes

* Pesticide registration and classification procedures, 40 C.F.R. Sect. 152.160-152.175 (2018).

† Responders might not have been required to take trainings listed as a condition of employment.

§ Worker protection, 40 C.F.R. Sect. 311.1 and 311.2 (2018).
